# Neonatal hypoglycemia screening in newborns from diabetic mothers - Arguments and controversies-

**Published:** 2014

**Authors:** A Stanescu, SM Stoicescu

**Affiliations:** * ”Dr. I. Cantacuzino” Clinical Hospital, Bucharest, Romania; ** ”Carol Davila” University of Medicine and Pharmacy, Bucharest; Neonatology Department, “Polizu” Clinical Hospital, Bucharest; “Alfred Rusescu” Institute for Mother and Child Care, Bucharest, Romania

**Keywords:** newborn, maternal diabetes, neonatal hypoglycemia, neonatal screening

## Abstract

The most important known metabolic complication of the newborns from diabetic mothers, including diabetes type 1 and 2 and gestational diabetes, is the postnatal hypoglycemia. If unrecognized and undiagnosed, hypoglycemia in this particular high-risk group can determine severe neurological lesions and even death, in a significantly higher proportion than those from the general population. The present paper brings arguments for the crucial importance of screening for post-natal hypoglycemia in the early hours after birth, and discusses the management strategies and the topics that still remain in debate.

Newborns from diabetic mothers, including diabetes type 1 and 2 and gestational diabetes, represent the group with the highest risk of developing symptomatic hypoglycemia in the immediate hours after birth. This metabolic risk is believed to be due to the relative fetal hyperinsulinism, manifested as a feedback mechanism for the balance of the high glucose levels induced by the maternal diabetes, and is particularly severe in those cases of poorly controlled maternal pre-existent diabetes with high levels of HbA1c [**1**,**2**]. 

Neonatal hypoglycemia screening in all newborns from diabetic mothers (IDM-infants from diabetic mothers) became mandatory from the first hour of life, because of the short- and long-term consequences of these episodes, independent of the type of maternal diabetes or of the initial good and balanced presentation at birth [**3**]. Compared to controls from non-diabetic mothers, the prevalence of hypoglycemic episodes in IDM is as high as 40% [**4**], and subsequently the risk of long-term neurological consequences, but also the immediate risk of convulsions, coma and even death is also significantly higher [**1**,**5**,**6**]. 

Episodes of hypoglycemia in IDM can persist up to one week after birth, with an increased frequency in the first 4 days of post-natal life, until the metabolic levels of endogenous insulin become balanced [**3**,**7**].

A consensus on the definition of hypoglycemia is still in debate in the recent literature, and also the current management of asymptomatic hypoglycemia and the necessities of exogenous carbohydrate intake are still under discussion, for the first hours of post-natal life [**4**,**8**,**9**].

Most studies describe hypoglycemia as levels under 30-50mg/dl (1,6-2,8 mmol/l) in the first 24 hours of post-natal life and 45-50mg/dl (2,5-2,8 mmol/L) after 24 hours [**3**,**4**,**9**]. The threshold levels of different studies for the definition of neonatal hypoglycemia are: under 45mg/d (2,5mmol/l)l (9), or 40 mg/dl (2,2mmo/l) or 36mg/dl(2 mmol/l) [**3**,**4**,**7**-**11**]. These variations are usually in the margins of the assay methods used or because of the actual source of the sample (plasma, serum, whole blood). The use of peripheral sampling with glucometers is efficient and user-friendly to screen for hypoglycemia, but for a certified diagnosis, a central sampling still needs to be done, as we know that the central levels are actually below the measurement of glucometers, and the relationship is not deductible from a formula calculus.

One important topic under discussion is the moment to start the screening and the frequency of measurements in the first hours of post-natal life. The algorithm advocated by the National Guideline on Neonatal Hypoglycemia Screening [**3**] is that, in the context of maternal diabetes, neonates should be screened immediately after birth and then at 30 min, 1 h, 2 hrs, 4 hrs, 8 and 12 hrs and at any moment when symptoms that suggest hypoglycemia occur [**3**]. Also, after the initiation of glucose-therapy, 30 minutes after the iv perfusion starts, and after every significant modification of the infused dose [**11**-**13**].

Cycles of glycemic control should start immediately at birth from the umbilical cord sampling at birth. A high level of glucose should trigger more attention as rebound hyperinsulinism could appear with subsequent severe hypoglycemia [**4**]. 

In order to prevent long-term neurological lesions, a quick response with glucose treatment should be initiated for all neonates who show symptoms of hypoglycemia. A bolus of 2ml/kg glucose 10% iv should be administered, followed by a continuous glucose perfusion of 6-8 mg/kg/min, in order to maintain glycemic levels over 40mg/dl (2,2 mmol/l) [**3**-**4**]. If, after the initiation of iv treatment, the glucose levels are still under 40mg/dl, treatment can be incremented with 2mg/kg/min or the concentration of the glucose solution to be changed to 12,5%, on a central way, until normal glucose levels are achieved [**5**,**9**,**12**,**13**]. Hydrocortisone can also be used as an adjuvant in very severe and resistant cases. The aim of the treatment is to obtain normal glucose levels for 12 hours and then a decrement of the supplements can be started with 1-2 mg/kg/min and the discontinuation of the iv supplementation when the enteral feeding is considered sufficient to maintain a correct glycemic control [**3**,**11**-**14**].

More debates concern the newborn with asymptomatic hypoglycemia. In these cases, the most important is the initiation of enteral feeding immediately after birth, during the first hour of life, breastfeeding or with assistance [**13**]. An alternative efficient method to keep normal glucose levels is to use dextrose-gel by oral massage in the first 48 hours of life.

The threshold levels at which iv glucose is judged necessary are also different between different protocols, variations are from 25mg/dl to 36mg/dl, or even as high as 40mg/dl, for those more protective. It is recommended to ample for glucose 30 minutes after the first breast-fed meal. In the first 4 hours of post-natal life, values under 25mg/dl in asymptomatic IDM, under enteral nutrition, will be resampled after 1 h. If the levels are still in the interval 25-40mg/dl, the newborn needs continuous monitoring. Values under 25mg/dl, at resampling, request the initiation of iv glucose therapy even in the absence of clinical symptoms [**3**].

The purpose of monitoring and therapy after the first 4 hours is to maintain glucose levels above 45 mg/dl in pre-prandial sampling, checked up to 12 hours of post-natal life. Under the threshold of 35mg/dl, in asymptomatic IDM, hourly checks are required. Borderline values between 35-45mg/dl trigger continuing monitoring. At any point in time values under 35mg/dl, initiate iv glucose therapy [**3**,**9**,**11**].

Monitoring can be discontinued when normal glucose levels are achieved for more than 12 hours, in the context of a clinically well newborn, who is enteral fed. 

## Conclusions

As the most important metabolic complication of the newborn from a diabetic mother, neonatal hypoglycemia should always be anticipated and screened for in high-risk patients, even in the absence of clinical symptoms, from the first moments of post-natal life. 

In this respect, all Neonatal Care Units should develop local protocols following the recommendations of the National Clinical Guide for the Screening and Treatment of Neonatal Hypoglycemia developed by consensus in 2010 and adopted by the Ministry of Health and the National Professional Boards [**4**].

It is very important to stress that the correct and early diagnosis and prevention of hypoglycemic episodes, in these fragile high-risk newborns, prevents long-term neurological effects and altered outcomes. 
